# Distribution of prolactin and its correlation with insulin resistance in women with polycystic ovary syndrome

**DOI:** 10.3389/fendo.2025.1674795

**Published:** 2025-10-01

**Authors:** Stefan Ghobrial, Robert Krysiak, Tal Goldstein, Antonella Patsch, Chiara Paternostro, Florian Heinzl, Rodrig Marculescu, Johannes Ott

**Affiliations:** ^1^ Clinical Division of Gynecologic Endocrinology and Reproductive Medicine, Medical University of Vienna, Vienna, Austria; ^2^ Department of Internal Medicine and Clinical Pharmacology, Medical University of Silesia, Katowice, Poland; ^3^ Department of Obstetrics and Gynecology, Landesklinikum Wiener Neustadt, Wiener Neustadt, Lower Austria, Austria; ^4^ Department of Obstetrics and Gynecology, Medical University of Vienna, Vienna, Austria; ^5^ Department of Laboratory Medicine, Medical University of Vienna, Vienna, Austria

**Keywords:** polycystic ovary syndrome, prolactin, insulin resistance, hyperprolactinemia, HOMA-IR

## Abstract

**Introduction:**

Polycystic ovary syndrome (PCOS) is often associated with insulin resistance (IR). The role of prolactin (PRL) in this context remains unclear, particularly across different PCOS phenotypes. The aim of this study was to investigate the distribution of PRL, as well as its correlation with basal IR in women with PCOS.

**Methods:**

200 women with PCOS, evenly distributed across phenotypes A-D and matched for age and body mass index (BMI) were retrospectively analyzed. PRL, Homeostasis Model Assessment of Insulin Resistance (HOMA-IR), sexual hormone binding globulin (SHBG), testosterone, and BMI were assessed. Correlation analysis and unsupervised clustering (based on PRL and HOMA-IR) were performed.

**Results:**

PRL levels were similar across phenotypes, but phenotype D had a significantly lower prevalence of HOMA-IR ≥ 2.5 (p = 0.032). PRL was inversely correlated with HOMA-IR in all groups (p < 0.05). Cluster analysis identified three distinct subgroups, independent of phenotype, differing significantly in both PRL and HOMA-IR.

**Conclusion:**

PRL is inversely associated with IR in PCOS, regardless of phenotype. Cluster analysis reveals metabolic subtypes not captured by current phenotype-based classification, suggesting potential for improved risk stratification.

## 1 Introduction

Polycystic ovary syndrome (PCOS) is the most frequent endocrine disorder in women of reproductive age with a prevalence of 6-15% when diagnosed according to the Rotterdam criteria (1). It is a complex endocrine disorder that not only affects the reproductive system, but is also associated with obesity, metabolic syndrome, type 2 diabetes mellitus and insulin resistance (IR), a condition where the body’s cells become less responsive to insulin, as well as other metabolic and cardiovascular risk factors (2–4). PCOS can be classified into four phenotypes depending on the expression of the potential symptoms after exclusion of other causes: clinical or biochemical hyperandrogenism (HA), chronic ovulatory dysfunction which manifests as oligo- or amenorrhea (OD) and polycystic ovarian morphology (PCOM). Phenotype A, the “complete phenotype”, includes all three criteria (HA + OD + PCOM). Phenotype B is diagnosed when HA and OD are present. The combination of HA and PCOM is typical for the “ovulatory” phenotype C and in women with the “normoandrogenic” phenotype D, OD and PCOM are found (2).

It has been shown that there are differences in metabolic risk between the four phenotypes. Phenotypes A, B and C seem to have a less favorable metabolic profile with a higher prevalence of metabolic syndrome and IR, compared to the “normoandrogenic” phenotype D (5–8). Notably, the “complete phenotype” A is associated with the least favorable metabolic outcomes (8, 9).

AMH (Anti-Müllerian Hormone) is elevated in women with PCOS compared to healthy women. Data on the distribution of AMH between the four phenotypes and its correlation with IR is quite heterogeneous. While some studies have found a positive correlation between AMH and IR measured by the Homeostasis Model Assessment (HOMA-IR) (10, 11), other studies have not been able to prove this (12). What the studies do agree on, however, is that the highest serum AMH and HOMA-IR levels are found in phenotype A. Moreover, a higher BMI also seems to correlate with IR (10–13). A positive correlation between a BMI > 25 kg/m^2^ and IR in women with PCOS has been demonstrated independently of their phenotype (11, 12). Further, IR and the resulting hyperinsulinemia as well as hyperprolactinemia (HPRL) inhibit the production of sex hormone binding globulin (SHBG) in the liver. This results in lower serum SHBG levels and thus the reduced binding of androgens to SHBG, leading to higher free testosterone concentrations (14).

Notably, HPRL is a condition of elevated prolactin (PRL), a hormone of the anterior pituitary gland, whose main physiological function is to initiate and maintain lactation (15). With a prevalence ranging from 0.4% in an unselected adult population to 17% in women with reproductive diseases, such as PCOS, it is also quite prevalent (15). Both disorders can be the cause of secondary amenorrhea, which is why the simultaneous diagnosis of HPRL and PCOS should not be a rare situation in women being examined for menstrual disorders (16).

The impact of PRL on metabolism is widely acknowledged. Both high and reduced PRL levels have been linked to IR in PCOS patients (17–19). Nevertheless, the influence of PRL on metabolism seems to be weight-dependent. Some studies suggest that mildly increased PRL levels (25–100 ng/mL) can be advantageous for metabolic health in obese PCOS patients (19). However, Bahceci et al. (17) demonstrated in their cohort of non-obese PCOS women that individuals with PRL levels exceeding 24 ng/mL already had an increased incidence of IR compared to the control group.

Due to the inconsistent data situation, the aim of this study was to investigate the distribution of PRL, as well as its correlation with basal HOMA-IR, in our cohort of 200 patients with PCOS. A specific focus was put on the differences and the distribution between the PCOS phenotypes. The study also intended to determine differences between the phenotypes regarding the correlations between PRL, BMI, HOMA-IR, SHBG and testosterone. Furthermore, the study aimed to evaluate an optimized cut-off for PRL concerning its association with pathological HOMA-IR≥ 2.5, considering the differences observed in the above-mentioned studies.

## 2 Materials and methods

### 2.1 Study design

This single-center, retrospective study was conducted at the Clinical Division of Gynecologic Endocrinology and Reproductive Medicine of the Medical University of Vienna, Austria, from January 2017 to December 2023. A total of 200 women with PCOS, aged 18–35 years were included. The study protocol was approved by the Ethics Committee of the Medical University of Vienna (IRB number 1298/2024).

PCOS was defined according to the revised Rotterdam criteria (1). In detail, hyperandrogenism was defined as the presence of hirsutism and/or a total testosterone level > 0.48 ng/mL (> 1.67 nmol/L), which is in accordance with the local normal ranges (20). Oligo-/anovulation was diagnosed based on the presence of oligo-/amenorrhea, i.e., a minimum cycle length ≥ 35 days in the last three months.

### 2.2 Patient population

Inclusion criteria were women aged 18–35 years with PCOS according to the revised Rotterdam criteria. Women who had received any PCOS-specific or HPRL-specific medication were excluded. Additional exclusion criteria included pregnancy, breastfeeding, known endocrine disorders other than PCOS (e.g., thyroid dysfunction, Cushing’s syndrome) or incomplete clinical or laboratory data.

From this patient cohort, 50 women per phenotype (A-D) were selected in order to obtain equally sized groups. This was achieved by 1:1 nearest-neighbor matching without replacement based on age (range ±1 year) and body mass index (BMI) (range ±1 kg/m²) until 50 patients per group were reached. To minimize bias, we verified balance of these matching variables between groups (see **Table 1**) and performed the main analyses (correlation analyses and unsupervised clustering) independently of phenotype.

**Table 1:** General patient characteristics in each PCOS phenotype group.

Parameters	A (n=50)	B (n=50)	C (n=50)	D (n=50)	p
Age (years)^1^	26 (23-28)	26 (23-29)	26 (23-29)	26 (23-29)	0.953
BMI (kg/m^2^)^1^	26.7 (22.0-32.6)	27.2 (21.6-32.3)	26.9 (21.9-32.5)	26.5 (22.1-32.5)	0.999
FSH (mIU/mL)^1^	5.5 (4.4-6.4)	5.3 (4.2-6.4)	5.6 (5.0-6.8)	5.6 (3.9-7.1)	0.305
LH (mIU/mL)^1^	12.9 (7.3-18.6)	13.4 (9.1-19.2)	11.8 (8.5-17.3)	10.4 (5.3-15.1)	0.109
LH: FSH ratio^1^	2.6 (1.9-3.1)	2.6 (2.0-3.5)	2.2 (1.5-2.9)	2.0 (1.2-2.8)	0.056
Testosterone (ng/mL)^1^	0.59 (0.47-0.71)^3^	0.61 (0.51-0.75)^3^	0.60 (0.52-0.75)^3^	0.37 (0.30-0.42)^4^	<0.001
DHEAS (µg/mL)^1^	3.41 (2.73-4.35)^3^	3.45 (2.78-4.47)^3^	3.86 (2.46-4.49)^3^	2.06 (1.64-2.74)^4^	<0.001
SHBG (nmol/L)^1^	37.5 (23.4-56.8)	40.2 (24.9-64.0)	43.7 (20.4-62.7)	44.0 (27.8-66.7)	0.497
AMH (ng/mL)^1^	9.9 (7.1-12.9)	6.1 (5.0-11.1)	7.9 (5.1-12.1)	7.7 (4.9-12.3)	0.249
Prolactin (pg/mL)^1^	12.9 (9.4-20.9)	11.7 (9.5-15.5)	12.0 (8.7-18.9)	11.2 (7.2-18.5)	0.622
HOMA-IR^1^	2.8 (1.8-3.6)	2.8 (1.8-3.6)	2.7 (1.9-3.5)	2.2 (1.4-3.1)	0.204
HOMA-IR >2.5^2^	30 (60.0)^3^	27 (54.0)^3^	28 (56.0)^3^	18 (36.0)^4^	0.032

Data are provided as ^1^ median (IQR) or ^2^ n (%); BMI, body mass index; FSH, follicle-stimulating hormone; LH, luteinizing hormone; DHEAS, dehydroepiandrosterone-sulfate; SHBG, sex hormone-binding globulin; AMH, anti-Müllerian hormone; HOMA-IR, Homeostasis Model Assessment of Insulin Resistance; IQR, interquartile range; *Statistical tests*, ANOVA for continuous variables; Chi-square test/Fisher’s exact test for categorical variables; *Post-hoc comparisons*: ^3^ significantly higher than group D (p < 0.05); ^4^ significantly lower than groups A-C (p < 0.05).

As a standard practice at our institution, all women with HPRL were recommended to undergo an MRI of the pituitary gland to rule out a prolactinoma before any HPRL-specific treatment, regardless of additional risk factors.

### 2.3 Parameters analyzed

The main outcome parameters were the distribution of PRL levels in our PCOS population, as well as its correlation with HOMA-IR diagnosed by blood samples on day 2 to 5 of the menstrual cycle.

The AKIM-software (SAP-based patient management system at the Medical University of Vienna) was used for data acquisition. Basic patient information, including age and BMI, was collected. To assess polycystic ovarian morphology (PCOM), an Aloka Prosound 6 ultrasound machine (Wiener Neudorf, Austria; frequency range 3.0 – 7.5 MHz) was used. PCOM is defined by a follicle number per ovary (FNPO) >20 in at least one ovary and/or an ovarian volume ≥10 ml or follicle number per section (FNPS) ≥ 10 in either ovary, which is consistent with international recommendations (21).

HPRL was defined as a PRL level > 25 ng/mL after serum precipitation with polyethylene glycol (PEG). This is to eliminate macroprolactin, a high molecular weight form of PRL that can cause artificially elevated PRL levels without accompanying symptoms. While macroprolactin is generally considered biologically inactive due to limited endothelial permeability, some reports suggest it may dissociate and release active monomeric PRL, potentially leading to symptoms in certain cases (16, 22, 23).

In addition to PRL, the following serological data were collected: serum levels of estradiol, luteinizing hormone (LH), follicle stimulating hormone (FSH), total testosterone, dehydroepiandrosterone-sulfate (DHEAS) and sexual hormone binding globulin (SHBG). Blood samples were obtained during the early follicular phase visit (cycle days 2 to 5). All serum parameters were determined at the Department of Laboratory Medicine, Medical University of Vienna, according to ISO 15189 quality standards. As reported previously (24), Cobas electrochemiluminescence immunoassays (ECLIA) were performed on Cobas e 602 analyzers (Roche, Mannheim, Germany) for the determination of serum PRL, estradiol, FSH, LH, testosterone, DHEAS, and SHBG.

The HOMA-IR, calculated as HOMA-IR=insulin (mU/L) * glucose (mg/dL)/405, ≥ 2.5 was used for the definition of IR according to previous studies (25, 26). Moreover, PCOS was classified into the four above-mentioned phenotypes.

### 2.4 Statistical analysis

Continuous variables are presented as medians with interquartile ranges (IQR), categorical parameters as numbers and frequencies. Differences between three groups were tested using analyses of variances (ANOVA) for numerical parameters and chi-square tests/Fisher’s exact tests for categorical parameters. Paired comparisons between three groups were performed by non-parametric ANOVA after rank transformation using the methodology suggested by Conover and Iman (27). Spearman rank correlations coefficients (r) were used to assess correlations between the parameters. This non-parametric method is suitable for monotonic relationships. Correlation coefficients range from -1 to 1, indicating the strength and direction of the correlation. Outliers in PRL levels were assessed using visual inspection (boxplots) and confirmed post-PEG precipitation to exclude macroprolactin interference. No data points were excluded unless clearly attributable to measurement or recording error. For these analyses, the IBM Statistical Package for Social Science software (SPSS 25.0; International Business Machines Corporation, New York, NY, USA) was used for all statistical tests. p-values < 0.05 were considered significant.

Patients were clustered using the k-means method (via the Hartigan and Wong algorithm), which partitions the cohort into k groups such that the sum of squares from the cluster centers to the patients are minimal. This partitioning was done with respect to regularized HOMA as well as PRL values.

The number of clusters was derived via majority vote; a total of 29 different approaches were used to calculate the optimal number of clusters (e.g. Elbow, Silhouette and Gap methods). A mode of 3 (14 votes) was identified and k was subsequently set to 3.

The analysis was conducted using R version 4.4.1 (R Core Team. 2024. R: A Language and Environment for Statistical Computing. Vienna, Austria: R Foundation for Statistical Computing. https://www.R-project.org/), along with the following packages: tidyverse v2.0.0 (Wickham, Hadley. 2023. Tidyverse: Easily Install and Load the Tidyverse. https://tidyverse.tidyverse.org), viridis v0.6.5 (Garnier, Simon. 2024. Viridis: Colorblind-Friendly Color Maps for r. https://sjmgarnier.github.io/viridis/), factoextra v1.0.7 (Kassambara, Alboukadel, and Fabian Mundt. 2020. Factoextra: Extract and Visualize the Results of Multivariate Data Analyses. http://www.sthda.com/english/rpkgs/factoextra) and parameters v0.22.2 (Lüdecke, Daniel, Dominique Makowski, Mattan S. Ben-Shachar, Indrajeet Patil, Søren Højsgaard, and Brenton M. Wiernik. 2024. Parameters: Processing of Model Parameters. https://easystats.github.io/parameters/).

Cluster analysis was followed by silhouette analysis to evaluate the separation distance between the resulting clusters. In detail, the silhouette plot displays a measure of how close each point in one cluster is to points in the neighboring clusters and thus provides a way to assess parameters like number of clusters visually. The average silhouette width has a range of -1 to +1. A value of +1 indicates that the sample is far away from the neighboring clusters, a value about 0 suggests that the sample is very close to the decision boundary between two neighboring clusters and negative values indicate that those samples might have been assigned to the wrong cluster. The number of clusters with the highest average silhouette width was considered optimal (28).

## 3 Results

### 3.1 Basic patient characteristics according to the PCOS phenotypes

Detailed patient characteristics are provided in **Table 1**. Due to the matching for age and BMI, these parameters were consistent across all groups. The hormonal profiles showed only little variations among the groups with group D, the “normoandrogenic” group, having significantly lower testosterone and DHEAS levels compared to the other groups. The number of women with a HOMA-IR≥ 2.5 was significantly lower in group D, whereas the median HOMA-IR values and PRL levels did not differ between the groups (**Table 1**).

### 3.2 Correlation analyses

To further explore relationships between HOMA-IR and the various parameters across the four phenotypic groups, Spearman rank correlations were calculated (**Table 2**). These revealed significant correlations between HOMA-IR and several parameters across the phenotypic groups. HOMA-IR was positively correlated with BMI in all groups. In contrast, HOMA-IR was negatively correlated with SHBG and PRL in all groups. Weak correlations were observed between BMI and PRL, with only Phenotype D showing a significant negative correlation, indicating that women with higher BMI in this group tend to have lower PRL levels. No significant correlations were found between HOMA-IR and testosterone.

**Table 2:** Correlation analyses.

Correlations	Phenotype A		Phenotype B		Phenotype C		Phenotype D	
	r	p	r	p	r	p	r	p
HOMA-IR vs BMI	0.627	<0.001	0.530	<0.001	0.606	<0.001	0.822	<0.001
HOMA-IR vs SHBG	-0.703	<0.001	-0.701	<0.001	-0.685	<0.001	-0.778	<0.001
HOMA-IR vs testosterone	0.253	0.086	0.234	0.106	-0.234	0.114	0.066	0.648
HOMA-IR vs prolactin	-0.302^1^	0.033^1^	-0.489^2^	<0.001^2^	-0.439^2^	<0.001^2^	-0.486^2^	<0.001^2^
BMI vs prolactin	0.115	0.427	-0.069	0.636	0.059	0.686	-0.235^3^	0.021^3^

HOMA-IR, Homeostasis Model Assessment of Insulin Resistance; BMI, body mass index; SHBG, sex hormone-binding globulin; *Statistical test*, Spearman rank correlation; ^1^ weak but significant negative correlation; ^2^ moderate negative correlation; ^3^ significant negative correlation observed only in phenotype D.

### 3.3 Cluster analysis

**Figure 1:**
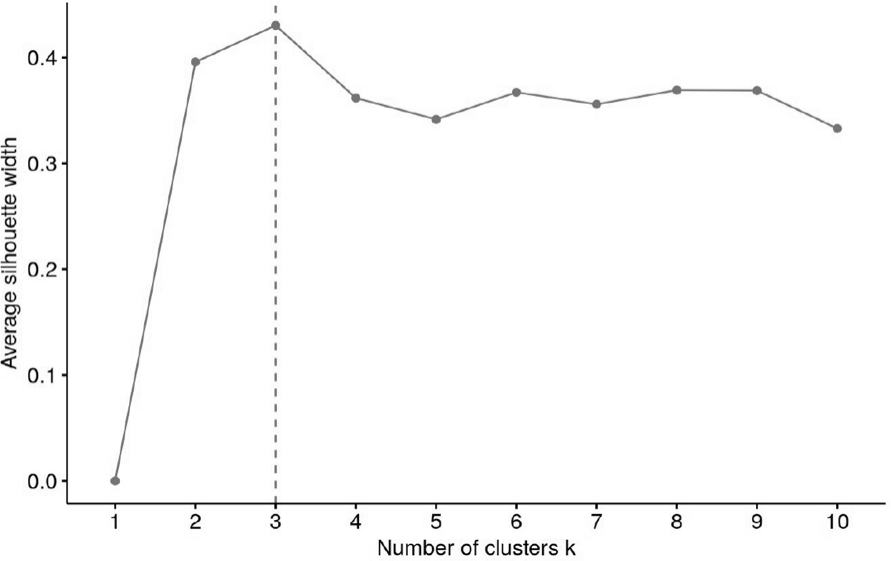
Evaluation of the optimal number of clusters using the silhouette method.

**Figure 2:**
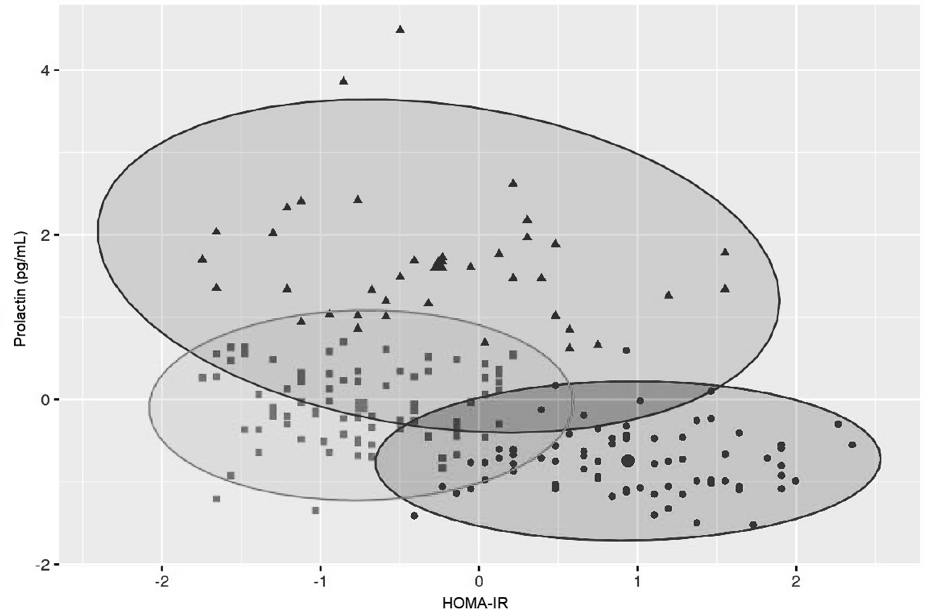
Cluster plot for prolactin and HOMA-IR.

Variables included in the cluster analyses were PRL and HOMA-IR. The optimal number of clusters with the highest silhouette width (0.4) was three (**Figure 1**). The clustering of individual patients by PRL and HOMA-IR is depicted in **Figure 2**. Details of comparison between the three clusters can be seen in **Table 3**. Concerning HOMA-IR and PRL, all three clusters differed significantly from each other according to non-parametric ANOVAs with *post hoc* Bonferroni correction. Patients in Cluster 1 revealed the highest median HOMA-IR levels as well as the lowest median PRL levels. In Cluster 2, the highest median PRL level of the three Clusters was found. The lowest median HOMA-IR was observed in Cluster 3.

**Table 3:** Comparison of groups according to cluster analysis.

Parameters		Cluster 1 (n= 77)	Cluster 2 (n= 39)	Cluster 3 (n= 84)	p
Age (years)^1^		26 (23-28)	25 (22-28)	27 (24-29)	0.113
BMI (kg/m^2^)^1^		29.2 (25.9-33.7)^4^	27.7 (22.8-34.0)^5^	23.4 (20.6-27.7)^4,5^	<0.001
FSH (mIU/mL)^1^		5.6 (4.9-6.7)	5.6 (4.5-6.9)	5.3 (4.2-6.5)	0.235
LH (mIU/mL)^1^		11.8 (8.4-15.9)	12.3 (7.4-16.7)	11.9 (6.7-18.7)	0.626
LH: FSH ratio^1^		2.2 (1.5-3.0)	2.4 (1.6-2.9)	2.6 (1.5-3.3)	0.516
Testosterone (ng/mL)^1^		0.59 (0.42-0.74)	0.53 (0.43-0.69)	0.47 (0.37-0.63)	0.078
DHEAS (µg/mL)^1^		3.30 (2.47-4.47)	3.62 (2.25-4.11)	2.87 (1.99-3.79)	0.048
SHBG (nmol/L)^1^		23.9 (19.3-33.2)^3,4^	47.2 (35.0-59.0)^3,5^	56.1 (43.7-77.7)^4,5^	<0.001
AMH (ng/mL)^1^		6.6 (5.0-12.1)	7.0 (4.8-9.4)	8.9 (6.0-13.3)	0.204
PCOS phenotype^2^	A	18 (23.4)	14 (35.9)	18 (21.4)	0.220
	B	22 (28.6)	4 (10.3)	24 (28.6)	
	C	21 (27.3)	13 (33.3)	16 (19.0)	
	D	16 (20.8)	8 (20.5)	26 (31.0)	
Prolactin (pg/mL)^1^		8.5 (6.3-10.1)^3,4^	25.2 (21.8-28.9)^3,5^	13.3 (10.9-16.5)^4,5^	<0.001
HOMA-IR^1^		3.7 (3.2-4.3)^3,4^	2.3 (1.7-3.1)^3,5^	1.8 (1.4-2.3)^4,5^	<0.001
HOMA-IR >2.5^2^		74 (96.1)	17 (43.6)	12 (14.3)	<0.001

Data are provided as ^1^ median (IQR) or ^2^ n (%); non-parametric ANOVAs with *post hoc* Bonferroni correction were performed; ^3^: significantly different between Cluster 1 and Cluster 2; ^4^: significantly different between Cluster 1 and Cluster 3; ^5^: significantly different between Cluster 2 and Cluster 3; BMI, body mass index; FSH, follicle-stimulating hormone; LH, luteinizing hormone; DHEAS, dehydroepiandrosterone-sulfate; SHBG, sex hormone-binding globulin; AMH, anti-Müllerian hormone; HOMA-IR, Homeostasis Model Assessment of Insulin Resistance; IQR, interquartile range.

## 4 Discussion

### 4.1 Phenotype D carries the lowest burden for IR

Our findings indicate that the different PCOS phenotypes are not uniformly associated with IR, with phenotype D exhibiting the lowest risk. This is consistent with previous studies that have found metabolic heterogeneity among PCOS phenotypes (29, 30). Phenotype D, characterized by the absence of hyperandrogenism, presents a more favorable metabolic profile compared to other phenotypes, primarily due to the absence of hyperandrogenic features, which are strongly correlated with increased IR and dyslipidemia (29, 31). Further, it showed a lower proportion of women with HOMA-IR ≥ 2.5 compared to the other phenotypes characterized by hyperandrogenism. This is also consistent with previous studies (30). Interestingly, median HOMA-IR levels did not differ significantly between the phenotypes. This suggests that threshold-based definitions of IR may uncover subtle but relevant group differences that mean values alone may not reveal.

This observation is consistent with the well-described relationship between hyperandrogenism and IR. Hyperinsulinemia stimulates the theca cells to increase androgen production and decreases at the same time hepatic SHBG synthesis, resulting in higher circulating levels of biologically active androgens. These androgens in turn aggravate IR by promoting central adiposity and impairing insulin signaling in peripheral tissues (3, 14). This feedback loop likely explains the less favorable metabolic profile of hyperandrogenic phenotypes A-C compared to the normoandrogenic phenotype D and is also further supported by recent evidence that highlights the higher metabolic risk of hyperandrogenic subgroups (32).

These findings highlight the importance of viewing PCOS not as a single, uniform condition but as a spectrum of related yet distinct disorders. Hyperandrogenic phenotypes appear more metabolically burdened, while normoandrogenic phenotype D may represent a less insulin-resistant subgroup.

### 4.2 No influence of PCOS phenotypes on interactions between HOMA-IR and PRL

Another aspect the study investigated was whether the different PCOS phenotypes influence the relationship between IR, measured by HOMA-IR, and serum PRL levels. Although PRL levels did not differ significantly between phenotypes, a consistent negative correlation between PRL and HOMA-IR across all phenotypes was observed. This suggests a potentially phenotype-independent inverse association between these two parameters. To the best of our knowledge, no previous study has directly examined this question. Existing research has either explored the relationship between PRL and IR without considering phenotype (33), or has compared IR across phenotypes without evaluating PRL levels (29, 30). This gap in the literature has left unanswered whether the relationship between PRL and HOMA-IR differs across PCOS phenotypes. By addressing this specific intersection, our study contributes new evidence suggesting that phenotypic classification does not significantly influence the PRL-IR relationship.

This inverse correlation may be explained by the physiological role of prolactin in metabolic regulation. At certain levels, PRL supports pancreatic β-cell proliferation and insulin secretion, enhances glucose uptake in peripheral tissues and contributes to glucose homeostasis (19). These beneficial effects might underlie the finding that women with relatively higher prolactin levels (although within the normal range) showed lower IR indices in our cohort. Conversely, very low PRL levels could represent a loss of this protective action, as seen in the subgroup with high HOMA-IR and low PRL (33).

### 4.3 Results of cluster analysis with new hypothesis

To further examine this potential relationship, an unsupervised, data-driven approach via cluster analysis was applied, including PRL and HOMA-IR as input variables. This identified three distinct clusters with significantly different profiles. Cluster 1 showed the highest IR and lowest PRL levels, while Cluster 2 had moderate HOMA-IR but the highest PRL levels. Cluster 3 had both low HOMA-IR and intermediate PRL.

What stood out most was that PRL, which didn’t differ across phenotypes, became a key discriminator in the clusters. This tells us that traditional PCOS classification systems might miss important hormonal-metabolic patterns. For example, Cluster 2 might represent a subgroup where PRL serves a compensatory role in early IR. In contrast, the lack of such a PRL response in Cluster 1, despite high HOMA-IR, could reflect a dysregulation that warrants further investigation. Cluster 3 seems more metabolically stable, neither PRL nor IR appeared particularly elevated.

Notably, the distribution of PCOS phenotypes across the three clusters wasn’t significant, which further supports the idea that these clusters reflect something different than what’s captured by phenotype alone. It also reinforces the value of combining classic endocrine markers like PRL with metabolic indices like HOMA-IR when trying to understand PCOS on a deeper level.

### 4.4 Study limitations and future directions

There are a few limitations to keep in mind when interpreting our findings. Firstly, PRL levels were measured only once. While this is common in clinical practice, it may not fully capture physiological variability in PRL concentrations. Secondly, because this was a retrospective study, cause-and-effect relationships couldn’t be established and there is always a chance of missing or incomplete data. Thirdly, IR was assessed using HOMA-IR, which was standard practice at the time, but according to the latest international PCOS guidelines (21), an oral glucose tolerance test (OGTT) would be the preferred method today due to its acceptable sensitivity, relatively low cost and its ability to diagnose diabetes. However, also the OGTT has limitations in accurately assessing IR. For research purposes, the hyperinsulinemic-euglycemic clamp remains the gold standard (34), though it is less feasible in large-scale or routine studies. Since neither OGTT or the hyperinsulinemic-euglycemic clamp technique nor multiple PRL measurements were routinely done in our cohort, this might have affected the precision of our evaluation of IR and PRL. Another, though probably less important drawback was the use of PEG precipitation to isolate monomeric PRL, which is widely accepted in clinical settings, although gel filtration chromatography offers greater specificity.

Another limitation is the use of electrochemiluminescence immunoassays (ECLIA) for androgen measurement. At the time of data collection, this was the routine method at our institution, whereas liquid chromatography-tandem mass spectrometry (LC-MS/MS) is now recommended as the gold standard (21). The limited sensitivity and precision of direct immunoassays in women with PCOS may partly explain why no significant correlation between HOMA-IR and testosterone was observed in our cohort, although such an association has been reported when free testosterone is measured by LC-MS/MS (35). At the same time, recent independent evaluations of the Roche ECLIA platform have demonstrated lower limits of quantification (LOQ) than initially indicated by the manufacturer, suggesting that its analytical performance is better than that of earlier immunoassays (36). Nevertheless, we fully recognize the superiority of LC-MS/MS and emphasize that future prospective studies should incorporate mass spectrometry-based methods for more accurate assessment of total and free testosterone in PCOS.

These limitations also highlight opportunities for future research. Prospective studies incorporating OGTT and where feasible, the clamp technique as the gold standard could provide a more accurate assessment of IR across PCOS phenotypes and help confirm the observed associations between PRL and metabolic markers. Additionally, larger and more diverse cohorts would be valuable to explore whether the cluster patterns identified in this study are reproducible and whether they have predictive value for clinical outcomes, such as the development of type 2 diabetes or cardiovascular disease. Finally, investigating the role of PRL not just as a marker but potentially as a modulator of metabolic health in PCOS could open up new avenues for risk stratification and personalized treatment strategies.

## 5 Conclusion

Our study highlights the limitations of phenotype-based classification in capturing the metabolic diversity of PCOS and suggests that PRL may play a more nuanced role in metabolic regulation than previously appreciated. The inverse association between PRL and IR appears to be consistent across phenotypes but becomes more pronounced when examined through cluster analysis. These findings open up new perspectives for refining risk stratification and may have implications for individualized therapeutic approaches in PCOS, particularly in identifying patients who may benefit from earlier metabolic interventions, even if their phenotype seems less risky on paper.

## Data Availability

The data analyzed in this study is subject to the following licenses/restrictions: The datasets analyzed for this study contain sensitive clinical information and are therefore not publicly available due to patient privacy and ethical restrictions. Data may be available from the corresponding author upon reasonable request and with appropriate institutional approvals. Requests to access these datasets should be directed to Johannes Ott, johannes.ott@meduniwien.ac.at.

## References

[1] Fauser BCJM Tarlatzis BC Rebar RW Legro RS Balen AH Lobo R. Consensus on women’s health aspects of polycystic ovary syndrome (PCOS): the Amsterdam ESHRE/ASRM-Sponsored 3rd PCOS Consensus Workshop Group. Fertility Sterility. (2012) 97:28–38. doi: 10.1016/j.fertnstert.2011.09.024, PMID: 22153789

[2] Lizneva D Suturina L Walker W Brakta S Gavrilova-Jordan L Azziz R. Criteria, prevalence, and phenotypes of polycystic ovary syndrome. Fertil Steril. (2016) 106:6–15. doi: 10.1016/j.fertnstert.2016.05.003, PMID: 27233760

[3] Diamanti-Kandarakis E Dunaif A. Insulin resistance and the polycystic ovary syndrome revisited: an update on mechanisms and implications. Endocr Rev. (2012) 33:981–1030. doi: 10.1210/er.2011-1034, PMID: 23065822 PMC5393155

[4] Gambineri A Patton L Altieri P Pagotto U Pizzi C Manzoli L. Polycystic ovary syndrome is a risk factor for type 2 diabetes: results from a long-term prospective study. Diabetes. (2012) 61:2369–74. doi: 10.2337/db11-1360, PMID: 22698921 PMC3425413

[5] Shroff R Syrop CH Davis W Van Voorhis BJ Dokras A. Risk of metabolic complications in the new PCOS phenotypes based on the Rotterdam criteria. Fertil Steril. (2007) 88:1389–95. doi: 10.1016/j.fertnstert.2007.01.032, PMID: 17462641

[6] Krentowska A Kowalska I. Metabolic syndrome and its components in different phenotypes of polycystic ovary syndrome. Diabetes Metab Res Rev. (2022) 38:e3464. doi: 10.1002/dmrr.3464, PMID: 33988288

[7] Borzan V Lerchbaum E Missbrenner C Heijboer AC Goschnik M Trummer C. Risk of insulin resistance and metabolic syndrome in women with hyperandrogenemia: A comparison between PCOS phenotypes and beyond. J Clin Med. (2021) 10:829. doi: 10.3390/jcm10040829, PMID: 33670546 PMC7922675

[8] Sachdeva G Gainder S Suri V Sachdeva N Chopra S. Comparison of the different PCOS phenotypes based on clinical metabolic, and hormonal profile, and their response to clomiphene. Indian J Endocrinol Metab. (2019) 23:326–31. doi: 10.4103/ijem.IJEM_30_19, PMID: 31641635 PMC6683693

[9] Altintas KZ Dilbaz B Cirik DA Ozelci R Zengin T Erginay ON. The incidence of metabolic syndrome in adolescents with different phenotypes of PCOS. Ginekol Pol. (2017) 88:289–95. doi: 10.5603/GP.a2017.0055, PMID: 28727126

[10] Wiweko B Indra I Susanto C Natadisastra M Hestiantoro A. The correlation between serum AMH and HOMA-IR among PCOS phenotypes. BMC Res Notes. (2018) 11:114. doi: 10.1186/s13104-018-3207-y, PMID: 29426356 PMC5807763

[11] Gupta M Yadav R Mahey R Agrawal A Upadhyay A Malhotra N. Correlation of body mass index (BMI), anti-mullerian hormone (AMH), and insulin resistance among different polycystic ovary syndrome (PCOS) phenotypes – a cross-sectional study. Gynecological Endocrinol. (2019) 35:970–3. doi: 10.1080/09513590.2019.1613640, PMID: 31081410

[12] Sahmay S Aydogan Mathyk B Sofiyeva N Atakul N Azemi A Erel T. Serum AMH levels and insulin resistance in women with PCOS. Eur J Obstetrics Gynecol Reprod Biol. (2018) 224:159–64. doi: 10.1016/j.ejogrb.2018.03.007, PMID: 29605710

[13] Siddiqui S Mateen S Ahmad R Moin S. A brief insight into the etiology, genetics, and immunology of polycystic ovarian syndrome (PCOS). J Assist Reprod Genet. (2022) 39:2439–73. doi: 10.1007/s10815-022-02625-7, PMID: 36190593 PMC9723082

[14] Plymate SR Matej LA Jones RE Friedl KE. Inhibition of sex hormone-binding globulin production in the human hepatoma (Hep G2) cell line by insulin and prolactin. J Clin Endocrinol Metab. (1988) 67:460–4. doi: 10.1210/jcem-67-3-460, PMID: 2842359

[15] Majumdar A Mangal NS. Hyperprolactinemia. J Hum Reprod Sci. (2013) 6:168–75. doi: 10.4103/0974-1208.121400, PMID: 24347930 PMC3853872

[16] Delcour C Robin G Young J Dewailly D. PCOS and Hyperprolactinemia: what do we know in 2019? Clin Med Insights Reprod Health. (2019) 13:1179558119871921. doi: 10.1177/1179558119871921, PMID: 31523136 PMC6734626

[17] Bahceci M Tuzcu A Bahceci S Tuzcu S. Is hyperprolactinemia associated with insulin resistance in non-obese patients with polycystic ovary syndrome? J Endocrinol Invest. (2003) 26:655–9. doi: 10.1007/BF03347025, PMID: 14594118

[18] Yang H Di J Pan J Yu R Teng Y Cai Z. The association between prolactin and metabolic parameters in PCOS women: A retrospective analysis. Front Endocrinol (Lausanne). (2020) 11:263. doi: 10.3389/fendo.2020.00263, PMID: 32477263 PMC7235367

[19] Macotela Y Ruiz-Herrera X Vázquez-Carrillo DI Ramírez-Hernandez G Martínez de la Escalera G Clapp C. The beneficial metabolic actions of prolactin. Front Endocrinol (Lausanne). (2022) 13:1001703. doi: 10.3389/fendo.2022.1001703, PMID: 36213259 PMC9539817

[20] Rotterdam ESHRE/ASRM-Sponsored PCOS consensus workshop group. Revised 2003 consensus on diagnostic criteria and long-term health risks related to polycystic ovary syndrome (PCOS). Hum Reprod. (2004) 19:41–7. doi: 10.1093/humrep/deh098, PMID: 14688154

[21] Teede HJ Tay CT Laven JJE Dokras A Moran LJ Piltonen TT. Recommendations from the 2023 international evidence-based guideline for the assessment and management of polycystic ovary syndrome. J Clin Endocrinol Metab. (2023) 108:2447–69. doi: 10.1210/clinem/dgad463, PMID: 37580314 PMC10505534

[22] Halbreich U Kinon BJ Gilmore JA Kahn LS. Elevated prolactin levels in patients with schizophrenia: mechanisms and related adverse effects. Psychoneuroendocrinology. (2003) 28 Suppl 1:53–67. doi: 10.1016/S0306-4530(02)00112-9, PMID: 12504072

[23] Shimatsu A Hattori N. Macroprolactinemia: diagnostic, clinical, and pathogenic significance. Clin Dev Immunol. (2012) 2012:167132. doi: 10.1155/2012/167132, PMID: 23304187 PMC3529459

[24] Hager M Hörath S Frigo P Koch M Marculescu R Ott J. Changes in serum markers of patients with PCOS during consecutive clomiphene stimulation cycles: a retrospective study. J Ovarian Res. (2019) 12:91. doi: 10.1186/s13048-019-0564-7, PMID: 31585548 PMC6777034

[25] Münzker J Hofer D Trummer C Ulbing M Harger A Pieber T. Testosterone to dihydrotestosterone ratio as a new biomarker for an adverse metabolic phenotype in the polycystic ovary syndrome. J Clin Endocrinol Metab. (2015) 100:653–60. doi: 10.1210/jc.2014-2523, PMID: 25387259

[26] Chen F Liao Y Chen M Yin H Chen G Huang Q. Evaluation of the efficacy of sex hormone-binding globulin in insulin resistance assessment based on HOMA-IR in patients with PCOS. Reprod Sci. (2021) 28:2504–13. doi: 10.1007/s43032-021-00535-0, PMID: 33721297

[27] Conover WJ Iman RL. Rank transformations as a bridge between parametric and nonparametric statistics. Am Statistician. (1981) 35:124–9. doi: 10.1080/00031305.1981.10479327

[28] Batool F Hennig C. Clustering with the average silhouette width. Comput Stat Data Analysis. (2021) 158:107190. doi: 10.1016/j.csda.2021.107190

[29] Zhao Y Fu L Li R Wang LN Yang Y Liu NN. Metabolic profiles characterizing different phenotypes of polycystic ovary syndrome: plasma metabolomics analysis. BMC Med. (2012) 10:153. doi: 10.1186/1741-7015-10-153, PMID: 23198915 PMC3599233

[30] Jamil AS Alalaf SK Al-Tawil NG Al-Shawaf T. A case-control observational study of insulin resistance and metabolic syndrome among the four phenotypes of polycystic ovary syndrome based on Rotterdam criteria. Reprod Health. (2015) 12:7. doi: 10.1186/1742-4755-12-7, PMID: 25595199 PMC4417246

[31] Myers SH Montanino Oliva M Nordio M Unfer V. PCOS phenotype focus: phenotype D under the magnifying glass. Arch Gynecol Obstet. (2024) 309:2307–13. doi: 10.1007/s00404-024-07408-2, PMID: 38502188

[32] Wen X Wang L Bai E. Metabolic characteristics of different phenotypes in reproductive-aged women with polycystic ovary syndrome. Front Endocrinol (Lausanne). (2024) 15:1370578. doi: 10.3389/fendo.2024.1370578, PMID: 39109080 PMC11300195

[33] Yang H Lin J Li H Liu Z Chen X Chen Q. Prolactin is associated with insulin resistance and beta-cell dysfunction in infertile women with polycystic ovary syndrome. Front Endocrinol (Lausanne). (2021) 12:571229. doi: 10.3389/fendo.2021.571229, PMID: 33716958 PMC7947819

[34] Kim JK. Hyperinsulinemic-euglycemic clamp to assess insulin sensitivity *in vivo* . Methods Mol Biol. (2009) 560:221–38. doi: 10.1007/978-1-59745-448-3_15, PMID: 19504253

[35] Yang Y Ding M Di N Azziz R Yang D Zhao X. Close correlation between hyperandrogenism and insulin resistance in women with polycystic ovary syndrome-Based on liquid chromatography with tandem mass spectrometry measurements. J Clin Lab Anal. (2019) 33:e22699. doi: 10.1002/jcla.22699, PMID: 30350882 PMC6818543

[36] Goreta S Zec I Bokulić A Marijančević D. Establishing LOQ for estradiol, LH, FSH and testosterone on Roche Cobas e801. Endocrine Abstracts (2023) 90:471. doi: 10.1530/endoabs.90.P471

